# Artificial Intelligence-Driven Prognosis of Respiratory Mechanics: Forecasting Tissue Hysteresivity Using Long Short-Term Memory and Continuous Sensor Data

**DOI:** 10.3390/s24175544

**Published:** 2024-08-27

**Authors:** Ghada Ben Othman, Amani R. Ynineb, Erhan Yumuk, Hamed Farbakhsh, Cristina Muresan, Isabela Roxana Birs, Alexandra De Raeve, Cosmin Copot, Clara M. Ionescu, Dana Copot

**Affiliations:** 1 Department of Electromechanics, System and Metal Engineering, Ghent University, Tech Lane Science Park 125, 9052 Ghent, Belgium; amani.ynineb@ugent.be (A.R.Y.); erhan.yumuk@ugent.be (E.Y.); hamed.farbakhsh@ugent.be (H.F.); isabelaroxana.birs@ugent.be (I.R.B.); claramihaela.ionescu@ugent.be (C.M.I.); dana.copot@ugent.be (D.C.); 2 Department of Control and Automation Engineering, Istanbul Technical University, Maslak, Istanbul 34469, Turkey; 3 Department of Automation, Technical University Cluj-Napoca, Memorandumului Street 20, 400114 Cluj, Romania; cristina.muresan@aut.utcluj.ro; 4 Fashion, Textiles and Innovation Lab (FTILab+), HOGENT University of Applied Science and Arts, Buchtenstraat 11, 9051 Ghent, Belgium; alexandra.deraeve@hogent.be (A.D.R.); cosmin.copot@hogent.be (C.C.)

**Keywords:** long short-term memory (LSTM), artificial intelligence, estimation, time-series forecasting, electrocardiogram, respiratory mechanics, continuous monitoring, lung function test, low-frequency oscillation technique

## Abstract

Tissue hysteresivity is an important marker for determining the onset and progression of respiratory diseases, calculated from forced oscillation lung function test data. This study aims to reduce the number and duration of required measurements by combining multivariate data from various sensing devices. We propose using the Forced Oscillation Technique (FOT) lung function test in both a low-frequency prototype and the commercial RESMON device, combined with continuous monitoring from the Equivital (EQV) LifeMonitor and processed by artificial intelligence (AI) algorithms. While AI and deep learning have been employed in various aspects of respiratory system analysis, such as predicting lung tissue displacement and respiratory failure, the prediction or forecasting of tissue hysteresivity remains largely unexplored in the literature. In this work, the Long Short-Term Memory (LSTM) model is used in two ways: (1) to estimate the hysteresivity coefficient η using heart rate (HR) data collected continuously by the EQV sensor, and (2) to forecast η values by first predicting the heart rate from electrocardiogram (ECG) data. Our methodology involves a rigorous two-hour measurement protocol, with synchronized data collection from the EQV, FOT, and RESMON devices. Our results demonstrate that LSTM networks can accurately estimate the tissue hysteresivity parameter η, achieving an $R^{2}$ of 0.851 and a mean squared error (MSE) of 0.296 for estimation, and forecast η with an $R^{2}$ of 0.883 and an MSE of 0.528, while significantly reducing the number of required measurements by a factor of three (i.e., from ten to three) for the patient. We conclude that our novel approach minimizes patient effort by reducing the measurement time and the overall ambulatory time and costs while highlighting the potential of artificial intelligence methods in respiratory monitoring.

## 1. Introduction

Non-invasive measurement of respiratory mechanics is pivotal for early diagnosis and management of lung diseases. Traditional techniques, such as spirometry, provide valuable insights but are often limited in their ability to offer continuous monitoring and require significant patient cooperation [1,2,3,4]. This creates a need for alternative methods capable of providing non-invasive, continuous, and reliable measurements. The Forced Oscillation Technique (FOT) lung function test has emerged as a promising alternative, providing non-invasive measurements of respiratory impedance by applying oscillatory pressure waves to the respiratory system under normal breathing conditions and recording the resultant airflow and pressure [5]. The result is a frequency response of the respiratory tissue, which can be used to characterize respiratory mechanical parameters, thereby assessing nominal or deviation values. When the response is measured above the breathing frequency, namely high-frequency oscillations (5–37 Hz), these are used to measure respiratory parameters such as resistance and reactance, offering insights into central airway properties and dynamic respiratory mechanics under different physiological conditions. In contrast, measurements closer to the breathing frequency in a lower range (0.2–2 Hz) provide valuable insights into lung viscoelastic properties and airway remodeling [6].

Significant research has been conducted to understand respiratory impedance and its clinical implications, leading to several non-invasive FOT monitoring systems. Low-frequency FOT (0.2–2 Hz) has shown effectiveness in indicating disease stages and predicting deterioration in conditions such as idiopathic pulmonary fibrosis [6,7,8,9]. Recent advancements have further enhanced our understanding of the mechanical behavior of lung tissues and peripheral airways [5,10,11]. Despite its potential, the continuous use of the FOT technique in clinical practice faces challenges, such as the impracticality of long-term measurements.

To address these challenges, artificial intelligence (AI) and machine learning algorithms have advanced research across the medical field. We speculate that AI algorithms can predict respiratory mechanics from smaller datasets, achieving high accuracy while minimizing the physical burden on patients. This study focuses on the use of Long Short-Term Memory (LSTM) networks, which are particularly effective in modeling time-series data, making them suitable for forecasting medical signals such as heart rate and respiratory parameters. Recent advancements highlight the effectiveness of AI in various clinical applications. For instance, AI algorithms have been used to predict the optimal timing for weaning patients from mechanical ventilation, achieving high accuracy and reducing ventilation duration [12]. LSTM networks, known for their proficiency in managing time-series data, have been applied to forecast emergency room visits for respiratory issues and predict respiratory rates from biosignals with high accuracy [13,14]. LSTM networks are increasingly used in predicting medical signals due to their effectiveness in modeling time-series data [15]. They adeptly handle both intra- and inter-series irregularities, which is crucial for accurately modeling complex medical signals such as heart rate [16,17]. This makes LSTMs particularly suitable for our task of predicting heart rate and the hysteresivity coefficient η parameter from respiratory trials. Despite significant advancements in the application of AI and deep learning to respiratory system analysis [18,19,20,21], the specific task of predicting or forecasting tissue hysteresis using AI remains unaddressed, positioning this study as a pioneering effort in the field.

In this study, we integrate low-frequency and high-frequency FOT measurements with continuous monitoring using the EQV LifeMonitor. The EQV is a non-invasive wearable sensor system that tracks physiological parameters such as heart rate, respiratory rate, and skin temperature. We aim to predict respiratory mechanics through continuous measurement, thereby reducing the physical burden on patients by leveraging AI-based approaches using LSTM networks. To achieve this, we propose two AI-based methodologies:

- The first approach aims to enhance the estimation of the hysteresivity coefficient η by employing continuously recorded heart rate data, thereby reducing the frequency of required measurements.
- The second approach focuses on forecasting η to anticipate respiratory issues, enabling early detection and intervention. Furthermore, FOT measurements can be used as daily calibration measurements.

Based on these predictions, clinicians can determine whether additional tests are warranted. For instance, if the AI model predicts a deterioration in respiratory mechanics, an additional FOT measurement can be conducted, and the data can be used to calibrate the forecasting model. This approach reduces the overall discomfort associated with repeated FOT measurements while ensuring accurate monitoring and timely clinical intervention.

This paper is organized as follows: In Section 2, the materials and methods used, including the FOT, Equivital physiological signal monitoring, and the measurement protocol, are introduced. Section 2 also presents the proposed AI-based methodologies for estimating and forecasting the hysteresivity coefficient η. In Section 3, the results of our study, including the performance metrics and a comparison of the estimation and forecasting approaches, are presented. Section 4 summarizes the key contributions and provides directions for future research. Appendix A provides a detailed explanation of the calculation of the η parameter.

## 2. Material and Methods

### 2.1. Forced Oscillation Technique

Normal quiet breathing during an FOT lung function test involves the contraction of the diaphragm, parasternal muscles, and scalene muscles. As the diaphragm moves downward during inhalation, it pulls the lower surfaces of the lungs with it. Exhalation follows when these muscles relax. Tissue structure and remodeling are linked to the heterogeneity or, alternatively, the degree of hysteresivity of the airways and alveoli. Changes in the elastic recoil of the lungs affect their stiffness, influencing the total lung volume and the pressure–volume relationship, which are indicators of lung disease [10].

Lung function can be assessed using the FOT by analyzing frequency responses resulting from oscillations at different frequencies [10,22]. This technique measures respiratory impedance, a complex variable where the real part represents the total resistance and the imaginary part shows the balance between the inertive and compliant (reactance) properties [23]. These impedance components, resistance and reactance, relate to morphological lung changes and are evaluated in the 5–37 Hz frequency range [24]. At frequencies below 5 Hz, the impedance is mainly influenced by the mechanical properties of peripheral airways and alveolar tissues.

The FOT is non-invasive, relatively effortless, and requires minimal cooperation from patients, making it particularly useful for pediatric or frail, critically ill patients. The frequency-dependent nature of impedance has been correlated with respiratory mechanical properties and can differentiate between restrictive and obstructive respiratory diseases. Additionally, recent advances in mathematical and anatomical modeling have revealed that frequency response impedance data provide insights into lung structure and function changes, which can be analyzed using mathematical models calibrated with real data [10].

**Figure 1:**
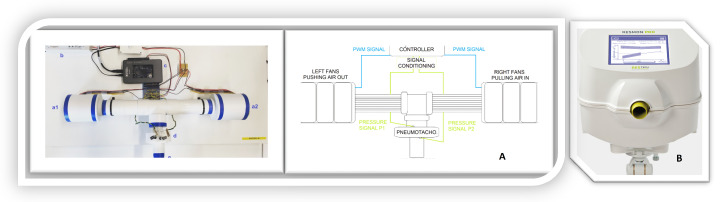
(**A**) Schematic of the 4P–FOT device’s principle of operation and related instrumentation. (**B**) RESMON Pro Full device.

In this study, both low-frequency and high-frequency devices are used to evaluate respiratory function. The low-frequency prototype device (4P-FOT), shown in Figure 1A, measures in the 0.2–2 Hz range, providing detailed insights into lung viscoelastic properties and airway remodeling [6]. The high-frequency commercial device (RESMON Pro Full), depicted in Figure 1B, operates in the 5–37 Hz range, offering a broader analysis of airway resistance and reactance and capturing dynamic respiratory mechanics changes under different physiological conditions [6].

### 2.2. Equivital Physiological Signal Monitoring

The EQV LifeMonitor is a wearable sensor system designed for continuous monitoring of physiological parameters, making it highly valuable for assessing respiratory function. This device is used in both clinical and research settings and provides real-time data on vital signs such as heart rate (HR), respiratory rate (RR), skin temperature (ST), and electrocardiogram (ECG) collected using electrode placements Lead I and Lead II, with these placements detailed in Appendix C. The EQV integrates multiple sensors to capture comprehensive physiological data: a tri-axis accelerometer for movement and position tracking, ECG electrodes for HR monitoring, and respiratory inductive plethysmography (RIP) bands to measure RR and breathing effort. This setup provides a detailed picture of an individual’s respiratory function. The EQV is user-friendly and comfortable for long-term monitoring, making it suitable for continuous patient monitoring, including remote applications.

Primarily used to monitor vital signs during exercise, the clinical potential of the EQV is still being explored. From a clinical perspective, the EQV could become an essential tool for monitoring respiratory diseases, such as Chronic Obstructive Pulmonary Disease (COPD). A recent study used the EQV device to monitor vital sign changes in COPD patients [25]. The study demonstrated the device’s ability to detect significant variations in respiratory rate and heart rate, which are critical indicators that could enable early intervention.

Several clinical trials have indicated that the FOT is a suitable technique for detecting and monitoring respiratory diseases. For example, Ref. [10] discussed the use of the low-frequency (4P-FOT) device to assess patients with asthma and COPD. Furthermore, both the FOT and RESMON devices have been used in clinical trials involving lung cancer patients, as it is well known that stage IV COPD can lead to lung cancer [5,6]. Ideally, one may correlate parameters from the FOT measurement with those continuously measured by the EQV, maximizing the richness of information gathered in real time from the subject.

**Figure 2:**
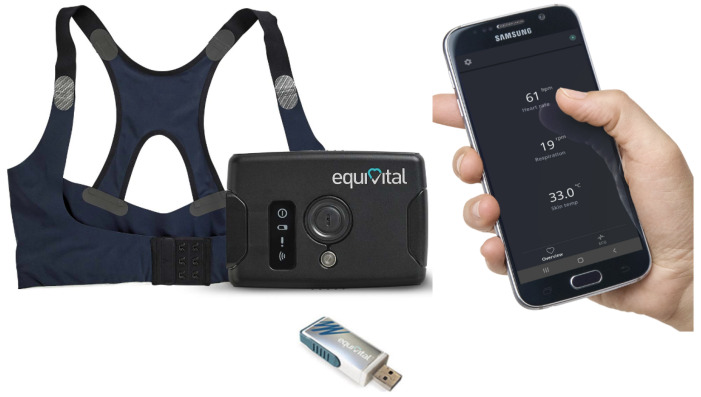
Schematic of the Equivital monitoring system.

In this study, the EQV sensor monitor, depicted in Figure 2, continuously collects data, including ECG heart rate, respiratory rate, and skin temperature, over a two-hour measurement period for each volunteer. Meanwhile, the FOT and RESMON devices are used alternately to measure respiratory parameters. Consequently, a synchronization step is essential to align the data from the EQV with the FOT measurements for detailed analysis. This synchronization allows for a comprehensive assessment by correlating continuously monitored physiological data with discrete respiratory measurements, ultimately aiming to improve the reliability of the estimated and forecasted values.

### 2.3. Measurement Protocol

**Figure 3:**
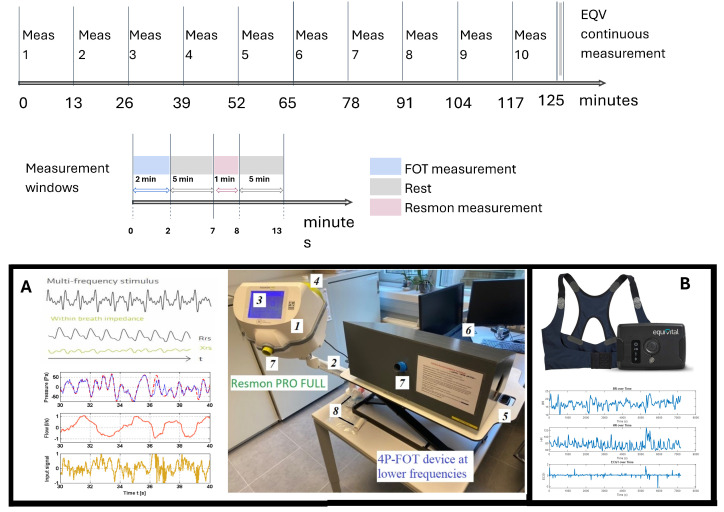
Upper graph: Illustration of the two-hour measurement protocol. Bottom: (**A**) Clinical setup comprising two FOT devices with their inputs and recorded signals. The RESMON Pro Full is a standalone device with two parts: the device (1) and an arm holder (2). It features a touchscreen display (3) for user interaction and a USB port (4) for data storage. The 4P-FOT device, used at lower frequencies, is mounted on an adjustable table (5) and is connected to a laptop (6) with built-in programs and a user interface. A single-use disposable mouthpiece (8) is connected to a slot (7) for each measurement. (**B**) The EQV real-time physiological signal monitoring sensor and the recorded signals.

A protocol has been designed to facilitate the integration of data recorded at varying sample intervals and distributed non-uniformly in time and frequency. The measurement protocol begins with a 2-min FOT measurement, followed by a 5-min rest period. This is succeeded by a 1-min RESMON measurement and another 5-min rest. This cycle repeats for 2 h. Figure 3 illustrates the timeline of this protocol over a two-hour period, divided into ten measurement sessions (Meas). Each session includes FOT and RESMON measurements interspersed with rest periods to maintain tidal breathing conditions, allowing for diaphragm muscle relaxation.

To synchronize the data extracted by the EQV with FOT measurements, the data are extracted at 2-min intervals, followed by an 11-min pause before the next extraction, repeating this pattern throughout the session. This method ensures accurate synchronization of EQV data with FOT measurements for comprehensive analysis. The timeline of the protocol starts at 0 min and extends to 125 min (2 h and 5 min), with each measurement window spanning 13 min. For Meas 10, the measurement ends without the final rest period, resulting in an 8-minute duration instead of the typical 13 min. The protocol is defined as follows:

- Two-minute FOT Measurement: Marked in blue, this phase starts each measurement session. Participants breathe normally while seated for 120 s.
- Five-minute Rest: Indicated in gray, this is a rest period.
- One-minute RESMON Measurement: Shown in pink, this phase involves using the RESMON device. Participants breathe normally while seated for 60 s.
- Five-minute Rest: Another rest period, depicted in gray.

### 2.4. Subjects

A cohort of 6 healthy volunteers participated in this study, following the two-hour measurement protocol. The biometric data for the subjects are presented in Table 1. All participants were informed about the measurement protocol and provided their written informed consent.

### 2.5. Proposed Estimation Algorithm

**Figure 4:**
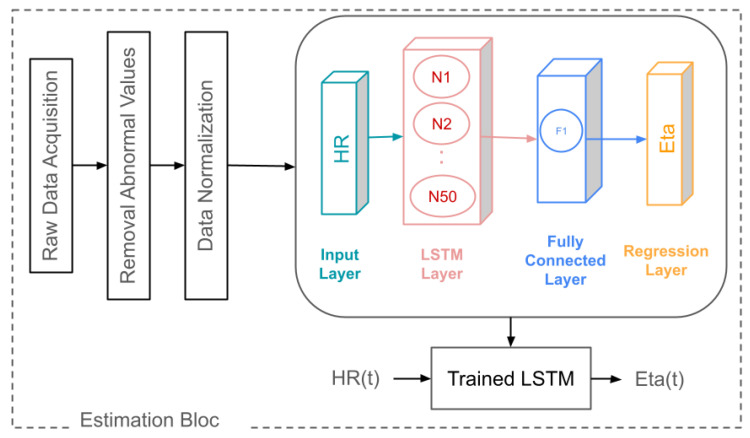
Schematic representation of the proposed AI algorithm for estimating η.

The primary aim of this study is to reduce the number of respiratory measurements using the FOT and RESMON devices from ten to three (specifically at intervals 1, 5, and 10). The goal is to estimate the η parameter, which measures tissue hysteresivity derived from respiratory impedance data. The input to the LSTM model is the continuous HR measured by the EQV sensor monitor, as depicted in Figure 4. The calculation of the hysteresivity coefficient η is detailed in Appendix A, and the LSTM architecture is described in Appendix B. Appendix D provides a detailed explanation of the estimation mechanism used to predict the η parameter.

To achieve this, the data are first preprocessed to ensure quality and consistency. Raw HR data are collected continuously using the EQV sensor monitor over a two-hour period. Following data preprocessing (outliers in the data are identified and smoothed), an LSTM network is trained to estimate the η values based on the HR data. The network architecture includes an input layer for the HR data, followed by an LSTM layer with 50 hidden units to process the time sequences. This LSTM layer captures the temporal dependencies in the HR data. A fully connected layer then maps the LSTM outputs to the desired η values, and a regression layer produces the final forecast. To enhance the model’s accuracy, we incorporate direct measurements at intervals 1, 5, and 10 as calibration points. These calibration points provide the model with reference data that help to fine-tune the estimation process.

During the training process, input sequences of HR data up to the current time point are prepared and used to train the LSTM network to predict the η values. For non-calibration points, the trained LSTM network uses the recorded HR data to make predictions. This approach enables the model to provide accurate estimates of η with fewer measurements, thereby reducing the overall physical and psychological burden.

### 2.6. Proposed Forecasting Algorithm

**Figure 5:**
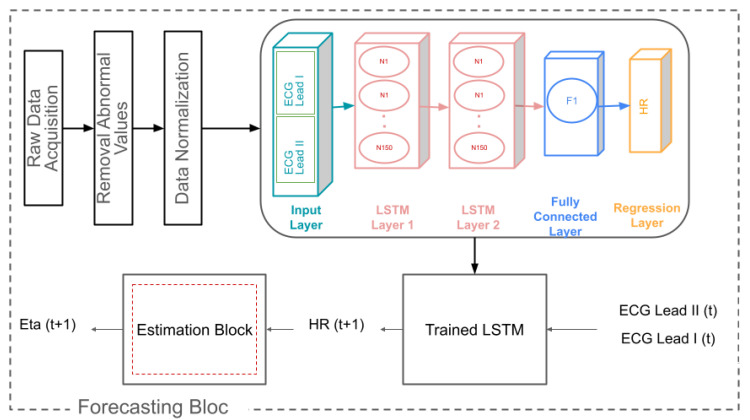
Schematic representation of the AI approach for estimating η.

Model-based forecasting properties in respiratory diseases, combined with treatment, have been recently proposed and successfully applied in clinical trials [6,26]. The second approach of this study focuses on forecasting the hysteresivity coefficient η rather than merely estimating it. This method, as presented in Figure 5 involves an additional step of forecasting the HR using ECG data before estimating η. The goal is to anticipate potential abnormalities or complications in respiratory mechanics, providing a proactive assessment framework. The changes in respiratory mechanics affecting the hysteresivity coefficient occur over a long period (months, years), and the proposed forecasting method introduced here can be used for monitoring the evolution of respiratory mechanics as a function of medication and revalidation. The forecasting model can incorporate the effects of drugs to indicate long-term outcomes and lead to optimal individualized management.

The forecasting process begins with raw ECG data from the EQV sensor monitor, specifically using data from the Lead I and Lead II placements. The collected ECG data undergo preprocessing to ensure quality and consistency. This preprocessing includes steps such as the removal of outliers and normalization of the data to standardize the input for the forecasting model, accounting for inter-individual biological variability.

Once the data are preprocessed, they are used as input for a forecasting model based on LSTM networks. The model architecture consists of two LSTM layers, each comprising 150 hidden units. These layers are designed to capture the temporal dependencies in the ECG data, which is essential for accurately predicting HR. Following the LSTM layers, a fully connected layer maps the learned features to the output size, corresponding to the forecasted HR values for the next time step. A regression layer is then used to compute the loss between the predicted and actual HR values during training.

The forecasted HR values serve as input to estimate η, following the estimation process described in Section 2.5. This integrated method allows for the continuous monitoring and prediction of respiratory mechanics. By predicting HR first, the model leverages the rich information contained in the ECG data to enhance the accuracy of η forecasting.

The prediction horizon is defined by the intervals between measurements, which are 11 min for the FOT device and 12 min for the RESMON device. This setup allows the model to forecast HR for the next time step, providing timely predictions that can inform clinical decisions. If the forecasted HR indicates potential respiratory issues, clinicians can decide to conduct additional FOT measurements to verify and address these issues promptly. This dual-step process, combining HR forecasting with η estimation, enables early detection and intervention for emerging respiratory complications. Appendix E provides a detailed explanation of the forecasting mechanism used to predict the η parameter.

All the computations in this study were conducted on a Dell workstation featuring an Intel^®^ Xeon^®^ Bronze 3204 processor operating at 1.90 GHz, with 64 GB of RAM, and running Windows 11 Pro for Workstations. The processor is manufactured by Intel Corporation, headquartered in Santa Clara, CA, USA. The data processing and analysis were performed using MATLAB 2022.

### 2.7. Performance Metrics

To assess the performance of the estimation results, the following performance metrics are employed:

(i) Mean Squared Error (MSE): MSE measures the average squared difference between the actual $y_{i}$ and predicted ${\hat{y}}_{i}$ values and is calculated as follows:
  (1) $$\mathrm{MSE}=\frac{1}{n}\sum_{i=1}^{n}{\left(y_{i}-{\hat{y}}_{i}\right)}^{2}.$$
(ii) Coefficient of Determination ($R^{2}$): $R^{2}$ quantifies the proportion of the variance in the dependent variable that is predictable from the independent variable(s) and is given by
  (2) $$R^{2}=1-\frac{\sum_{i=1}^{n}{\left(y_{i}-{\hat{y}}_{i}\right)}^{2}}{\sum_{i=1}^{n}{\left(y_{i}-\bar{y}\right)}^{2}},$$
  where $\bar{y}$ is the mean of the observed values.

The MSE provides insights into the accuracy of the estimates, with lower values indicating better performance. $R^{2}$ ranges from 0 to 1, where a higher $R^{2}$ signifies a better fit of the model to the data. The analysis includes both MSE and $R^{2}$ values to assess the effectiveness of the estimation and forecasting approaches.

## 3. Results

**Figure 6:**
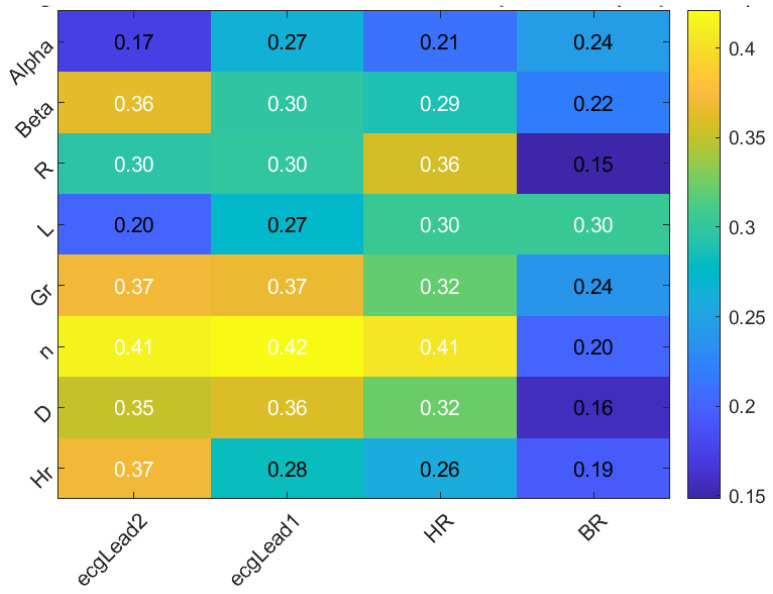
The average correlation between impedance model parameters and physiological parameters measured by the EQV sensor across all individuals.

The significance of correlation rates between inputs and outputs plays an important role in evaluating estimation accuracy within AI, prediction, and forecasting domains. High correlation rates can simplify neural network architectures, thereby enhancing their capacity to model and predict accurately [27,28]. Figure 6 illustrates the average correlation matrix between the impedance model parameters and the physiological parameters measured by the EQV sensor across six volunteers.

The matrix reports notable correlations between certain impedance model parameters and physiological measures. The parameter η shows moderate correlations with ECG Lead I, ECG Lead II, and HR, with values of 0.42, 0.41, and 0.41, respectively, indicating a stable relationship across all individuals. Additionally, *D* and $G_{r}$ demonstrate significant correlations with ECG Lead I and ECG Lead II, with values exceeding 0.35, suggesting possible interactions between these parameters. For details on these model parameters, see Appendix A. These moderate correlations across multiple individuals suggest potential interdependencies between the impedance model parameters and the physiological measures.

The strong correlation of η with ECG Lead I and ECG Lead II suggests that η is closely related to the electrical properties of cardiac activity. This relationship indicates that variations in cardiac cycles significantly influence impedance, reflecting changes in heart function, and could serve as an additional estimator for lung disease. The notable correlations between HR and impedance parameters, such as η, further suggest that heart activity can be an additional indicator of lung condition. This reasoning justifies the use of ECG Lead I, ECG Lead II, and HR as inputs for the LSTM model to estimate or forecast η.

### 3.1. Estimation Approach Results

**Figure 7:**
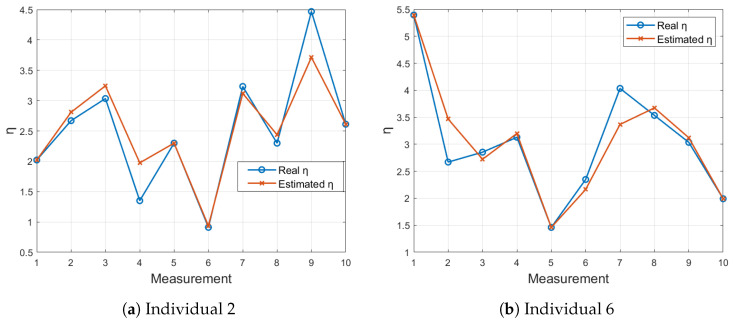
Comparison of LSTM-estimated η values with actual η values for 10 measurements using the FOT device.

The results depicted in Figure 7 show a comparison between the LSTM-estimated η values and the actual η values calculated from the FOT device parameters. For Individual 2 (78% fit), as presented in Figure 7a, the estimated values exhibit a deviation from the actual values in measurements 4 and 9.

This indicates that the model struggled to accurately predict the η values for this subject. Conversely, for Individual 6 (with an excellent fit of 85%), as shown in Figure 7a, the LSTM model closely follows the actual η values. This observation suggests robust predictive capability for this subject, revealing the potential of using LSTM models for reliable estimation of η values.

**Figure 8:**
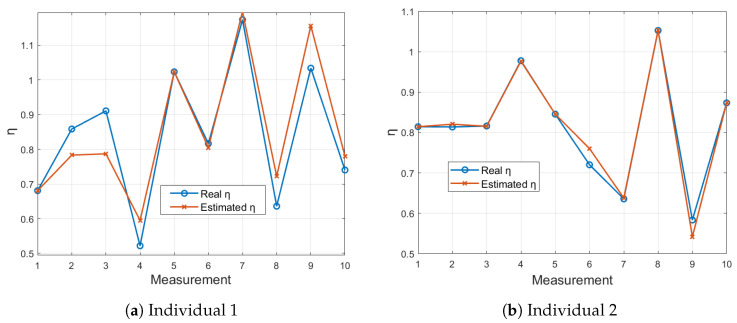
Comparison of LSTM-estimated η values with actual η values for 10 measurements using the RESMON device.

Figure 8 presents a similar comparison using the RESMON device. In Figure 8a, the results for Individual 1 show a close alignment between the estimated and actual η values, indicating strong model performance. Figure 8b for Individual 2, shows an almost perfect match between the estimated and actual values.

The performance metrics presented in Table 2 demonstrate the variability in model accuracy across various individuals for both the FOT and RESMON devices. For the FOT device, the MSE and $R^{2}$ values indicate that Individual 2 (MSE: 0.106; $R^{2}$: 0.781) and Individual 6 (MSE: 0.296; $R^{2}$: 0.851) show the highest predictive accuracy, with the model closely aligning with the actual η values. Conversely, Individual 3 (MSE: 1.405; $R^{2}$: 0.472) exhibits the poorest performance, with significant deviations between the estimated and actual values.

Similarly, the RESMON device results highlight Individual 2 (MSE: 0.091; $R^{2}$: 0.861) as having the most accurate predictions, followed by Individual 1 (MSE: 0.201; $R^{2}$: 0.802). Individual 3 (MSE: 1.405; $R^{2}$: 0.341) again shows the least accurate predictions. The *p*-values across all individuals for both devices indicate that the differences between the predicted and actual η values are not statistically significant, supporting the robustness of the LSTM model’s predictions.

### 3.2. Forecasting Approach Results

**Figure 9:**
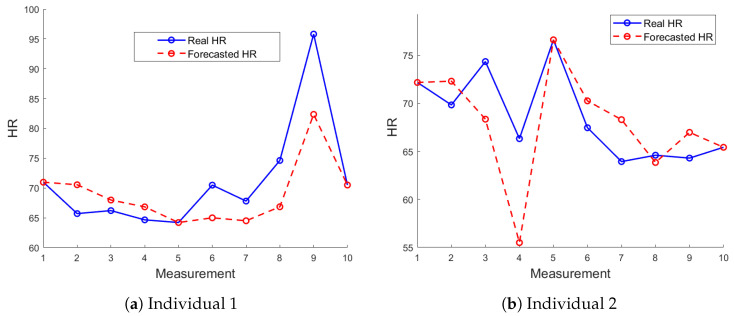
Comparison of LSTM-forecasted HR values with actual HR values for 10 measurements using the FOT device.

In Figure 9, the forecasted HR using the LSTM model is compared to the actual HR values over ten measurements with the FOT device. For Individual 2, as shown in Figure 9b, the forecasted HR values exhibit moderate accuracy. For Individual 1, as shown in Figure 9a, the forecasted HR closely follows the profile of the actual values, demonstrating the model’s ability to effectively capture and predict HR trends for this subject.

**Figure 10:**
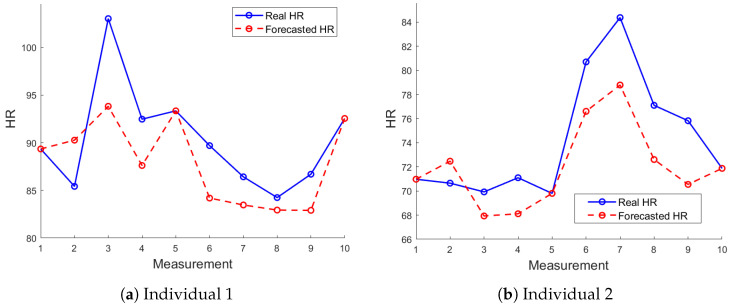
Comparison of LSTM-forecasted HR values with actual HR values for 10 measurements using the RESMON device.

Figure 10 illustrates the LSTM-forecasted HR compared to the actual HR values recorded during RESMON device monitoring. Figure 10a,b present the results for Individual 1 (62% fit) and Individual 2 (56% fit), respectively. Both figures show a good degree of accuracy, with the forecasted HR values closely following the actual values.

**Figure 11:**
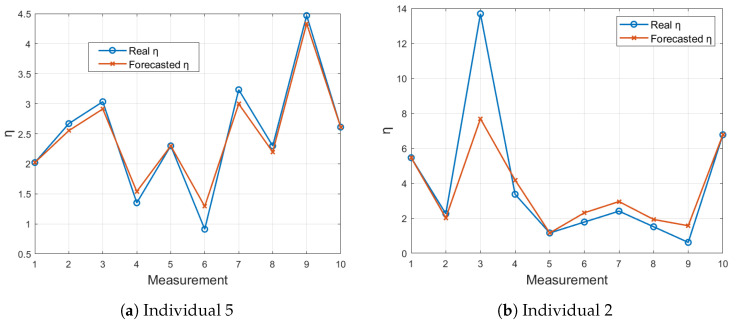
Comparison of LSTM-forecasted η values with actual η values for 10 measurements using the FOT device.

In Figure 11, the forecasted η values using the LSTM model are compared to the actual η values for the FOT device. Individual 2, as shown in Figure 11b, shows moderate prediction accuracy with noticeable deviations. In contrast, Individual 5, as shown in Figure 11a, exhibits a closer alignment between the forecasted and actual values, demonstrating the effectiveness of the proposed algorithm.

**Figure 12:**
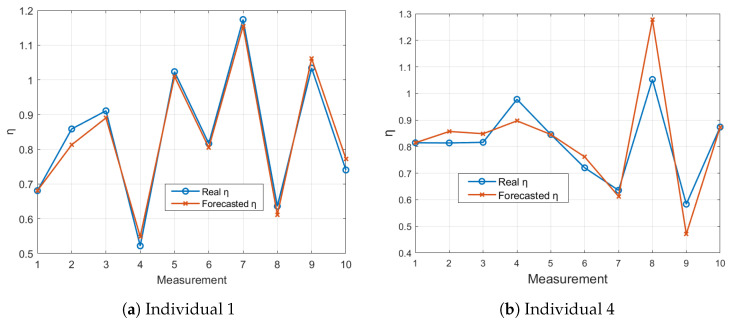
Comparison of LSTM-forecasted η values with actual η values for 10 measurements using the RESMON device.

Figure 12 presents the LSTM-forecasted η values compared to the actual η values for the RESMON device. Both Individual 1 and Individual 4 show excellent alignment in their respective figures (Figure 12a,b). These findings suggest that the LSTM model is well suited for the proactive assessment and early detection of potential abnormalities in respiratory parameters.

The performance metrics for individuals using the FOT and RESMON devices are outlined in Table 3. For the FOT device, Individual 5 (MSE: 1.122; $R^{2}$: 0.832) demonstrates the highest predictive accuracy, as indicated by a low MSE and high $R^{2}$. Conversely, Individuals 1 (MSE: 3.149; $R^{2}$: 0.563), 4 (MSE: 3.487; $R^{2}$: 0.427), and 6 (MSE: 3.690; $R^{2}$: 0.392) show higher MSE values and lower $R^{2}$ scores, indicating poorer model performance in predicting η values.

When examining the RESMON device results, Individual 1 (MSE: 0.528; $R^{2}$: 0.883) exhibits the best performance, closely followed by Individual 6 (MSE: 0.692; $R^{2}$: 0.724). In contrast, Individuals 3 (MSE: 1.406; $R^{2}$: 0.531) and 2 (MSE: 1.057; $R^{2}$: 0.588) exhibit relatively higher errors and lower correlation coefficients. The *p*-values for all individuals across both devices again suggest that the differences between the predicted and actual η values are not statistically significant, indicating that the model predictions are reliable.

To forecast HR data, two ECG leads were used as inputs; however, standard monitors typically measure ECG data at a single site, often using only Lead I or Lead II [29]. Our data across six individuals demonstrated that it is possible to predict one ECG lead signal from another, indicating that even monitors using a single lead can effectively apply the predictive approach proposed in this study. This suggests that our method can be broadly applicable, providing reliable estimates of ECG signals regardless of the number of leads used.

LSTM was used to predict ECG Lead II signals based on ECG Lead I signals. The network architecture included two LSTM layers, each comprising 100 hidden units and configured to output sequences. Following the LSTM layers, a fully connected layer was employed to map the learned features to the output size of 1, which corresponds to the predicted ECG signal. Finally, a regression layer was added to compute the loss between the predicted and actual ECG signals during training.

The network was trained using the Adam optimizer with a maximum of 200 epochs. The data were split into training and validation sets in a 70:30 ratio, ensuring that the model was trained on a representative subset of the data and validated on unseen data.

**Figure 13:**
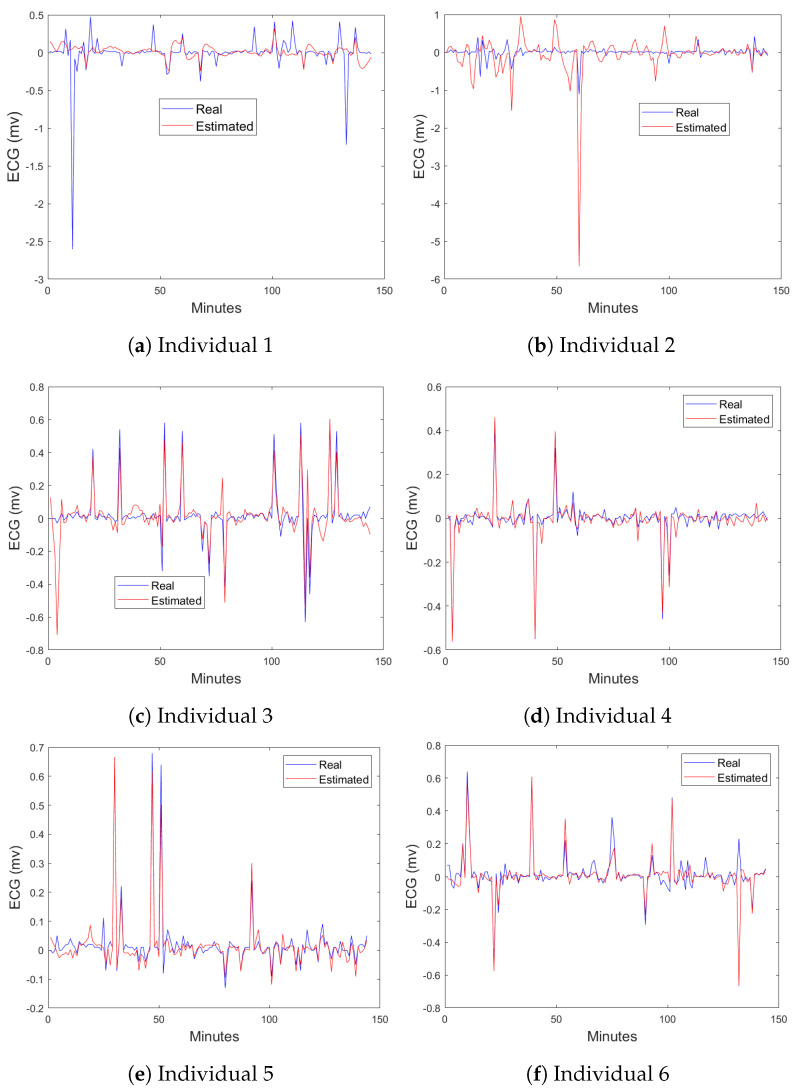
Comparison of real and estimated ECG Lead II signals for six volunteers using the EQV sensor. The estimated signal is in red, and the real signal is in blue.

Figure 13 presents a comparison between the real and estimated ECG Lead II signals for six volunteers using the EQV sensor.

Figure 13a shows the real versus estimated ECG Lead II signals for Individual 1. The estimated signal (red) does not align closely with the real signal (blue), indicating a poor fit (39.1%). This suggests that the model struggled to capture the temporal patterns and fluctuations in the ECG signals, which were relatively atypical or outlier-like for this subject.

Figure 13b depicts the comparison for Individual 2. Similar to Individual 1, the estimated signal shows significant discrepancies compared to the real signal, especially during the peaks and troughs, indicating another instance of poor fit (33.7%).

Figure 13c–f show the real and estimated signals for Individuals 3 to 6, respectively. For these volunteers, the estimated signals closely follow the real signals, with only minor deviations. The model effectively captures the key features and temporal patterns of the ECG signals, indicating a very good fit (above 60%) and suggesting potential for capturing real-time anomaly detection. This demonstrates the model’s capability to predict ECG Lead II signals based on ECG Lead I inputs with high accuracy for these subjects.

## 4. Discussion

As initially speculated, the findings of this study demonstrate the effectiveness of using LSTM networks to estimate and forecast the hysteresivity coefficient η from respiratory impedance data, achieving the goals of reducing the number of required measurements and predicting future complications. The dual research objective involves, firstly, estimating the tissue hysteresivity η using HR data collected continuously by the EQV sensor monitor and, secondly, forecasting HR from ECG data to anticipate future η values. The proposed multivariate and integrated method enhances the accuracy and reliability of η estimates, with the secondary outcome of reducing the overall physical and psychological burden by significantly reducing the number of necessary measurements from ten to three. In fact, the reverse statement of this conclusion holds: given the reduced number of measurements, reliable estimates and forecasting values are obtained from multivariate sensor data.

### 4.1. Findings

The results demonstrate that LSTM networks can accurately estimate η values using HR data. Specifically, for the individuals tested with the FOT device, the model achieved high predictive accuracy for Individuals 2 and 6, as evidenced by their low Mean Squared Error (MSE) and high Coefficient of Determination ($R^{2}$) values. Similarly, for the RESMON device, Individuals 1 and 2 exhibited strong alignment between the estimated and actual η values, indicating robust model performance.

This study also highlights the LSTM model’s capability to forecast HR values based on electrocardiogram (ECG) data, which are then used to predict η. This dual-step forecasting algorithm proved effective, with Individuals 5 and 2 showing close alignment between the forecasted and actual η values when using the FOT device. For the RESMON device, Individuals 1 and 4 demonstrated excellent alignment, suggesting the model’s potential for early detection of alterations in respiratory mechanics.

The ability to estimate and forecast η using fewer measurements has broad and significant clinical implications. By reducing the number of required FOT and RESMON lung function test measurements, not only are costs minimized, but discomfort and burden are also reduced, which is particularly beneficial for those with severe respiratory conditions. Moreover, the integration of AI-driven methods for continuous monitoring allows for proactive management of respiratory healthcare. Clinicians can rely on AI predictions to determine the necessity of additional FOT measurements, thereby optimizing the diagnostic process and potentially improving clinical outcomes.

Our study introduces a novel approach by not only estimating but also forecasting tissue hysteresis using LSTM networks. Previous studies have focused on classification and predicting current respiratory system states or stability [30,31]. This proactive forecasting of tissue hysteresis sets our study apart by providing early intervention opportunities in respiratory care, which were not previously possible. Additionally, while other studies have successfully forecasted variables in respiratory systems [32,33], our focus on tissue hysteresis represents a unique and valuable contribution to the field, offering a new dimension of predictive capability in respiratory health management.

### 4.2. Clinical Application

This study demonstrates the potential of LSTM networks to estimate and forecast the hysteresivity coefficient η using continuous HR data from the EQV sensor monitor, combined with periodic FOT and RESMON measurements. The forecasting interval proposed here is short and can be applied to monitoring in real-time onset of respiratory patterns and mechanics, e.g., sleep studies, obstructive sleep apnea, histaminic responses, asthma triggers, post-surgery lung resection monitoring, etc. It can be easily extended to longer intervals to evaluate the forecasting of tissue mechanics alterations as respiratory disease progression occurs or to monitor treatment efficacy and determine optimal drug dosage regimes. Furthermore, integrating the proposed method into ambulatory care systems, particularly for elderly individuals, holds significant promise. By leveraging LSTM networks within remote health monitoring systems, it becomes feasible to provide continuous, real-time assessment of respiratory and cardiac health in older adults. Such systems could be deployed in home environments, reducing the need for frequent hospital visits and enabling the early detection of potential issues before they escalate into emergencies.

### 4.3. Limitations

This study demonstrates promising results in using LSTM networks to estimate and forecast the hysteresivity coefficient η from respiratory impedance data. However, several limitations must be acknowledged. One significant limitation is the small sample size, which included only six healthy volunteers. This limited and homogeneous sample may not fully capture the variability present in a broader population, particularly those with respiratory diseases. Consequently, the findings may not be easily generalizable. To improve model generalizability, future research should focus on expanding the sample size to include a more diverse cohort, encompassing both healthy individuals and patients with various respiratory conditions. This would help in testing the robustness of the model and ensuring its applicability across different patient populations.

Additionally, the integration of data from different devices, such as the FOT and RESMON devices, posed synchronization challenges that could impact the accuracy of the results. Although these challenges were managed in this study, further work is needed to refine the synchronization process, particularly when integrating more complex or additional variables. For example, integrating Electrical Impedance Tomography (EIT) [24,34], a technique that provides real-time imaging of lung function, and Respiratory Rate Variability (RRV) [35] could offer more comprehensive insights into respiratory mechanics. These parameters would complement existing data by providing more detailed information on lung function and respiratory patterns, thereby enhancing both the predictive accuracy and clinical relevance of the model. Moreover, the implementation of real-time monitoring and predictive analytics in clinical settings also needs further investigation to assess their practical feasibility, integration with existing healthcare infrastructure, and overall impact on patient outcomes.

## 5. Conclusions

This study demonstrates the ability of the LSTM model to estimate and forecast the hysteresivity coefficient η from respiratory impedance data by integrating continuous HR data from the EQV sensor monitor with periodic FOT and RESMON lung function test data. By employing AI-based approaches to reduce the number of required respiratory measurements, this study highlights significant potential for minimizing costs and effort without sacrificing accuracy. The variability in model performance across different volunteers underscores the importance of personalized medicine, suggesting that tailored healthcare solutions can optimize treatment outcomes. However, this study is limited by the small sample size and the homogeneity of the cohort, which consisted solely of healthy individuals. Additionally, the use of a single AI model (LSTM) and the lack of comparison with other approaches limit the generalizability of the findings. Future research should validate these methods in larger, more diverse populations and explore the use of multiple AI models to enhance the robustness and applicability of the results in clinical settings, aiming to improve healthcare in respiratory medicine.

## Figures and Tables

**Table 1:** Biometric parameters of the measured subjects.

ID	Age (Years)	Weight (kg)	Height (cm)	BMI (kg/m^2^)
1	37	53	165	19
2	40	85	180	26
3	28	62	160	24
4	35	78	172	26
5	29	90	179	28
6	28	51	163	19

**Table 2:** Performance metrics of the LSTM model for estimating the hysteresivity coefficient η using FOT and RESMON devices across various individuals.

	FOT Device			RESMON Device		
Volunteer	MSE	$R^{2}$	* p * -Value	MSE	$R^{2}$	* p * -Value
1	0.321	0.598	0.654	0.201	0.802	0.884
2	0.106	0.781	0.948	0.091	0.861	0.992
3	1.405	0.472	0.526	1.405	0.341	0.745
4	0.116	0.795	0.982	0.254	0.786	0.976
5	0.833	0.805	0.822	0.262	0.824	0.865
6	0.296	0.851	0.782	0.354	0.798	0.770

**Table 3:** Performance metrics of the LSTM model for forecasting the hysteresivity coefficient η using FOT and RESMON devices across various individuals.

	FOT Device			RESMON Device		
Volunteer	MSE	$R^{2}$	* p * -Value	MSE	$R^{2}$	* p * -Value
1	3.149	0.563	0.136	0.528	0.883	0.956
2	0.129	0.727	0.973	1.057	0.588	0.877
3	0.838	0.726	0.839	1.406	0.531	0.679
4	3.487	0.427	0.181	0.807	0.622	0.625
5	1.122	0.832	0.668	1.174	0.669	0.656
6	3.690	0.392	0.343	0.692	0.724	0.959

**Figure A1:**
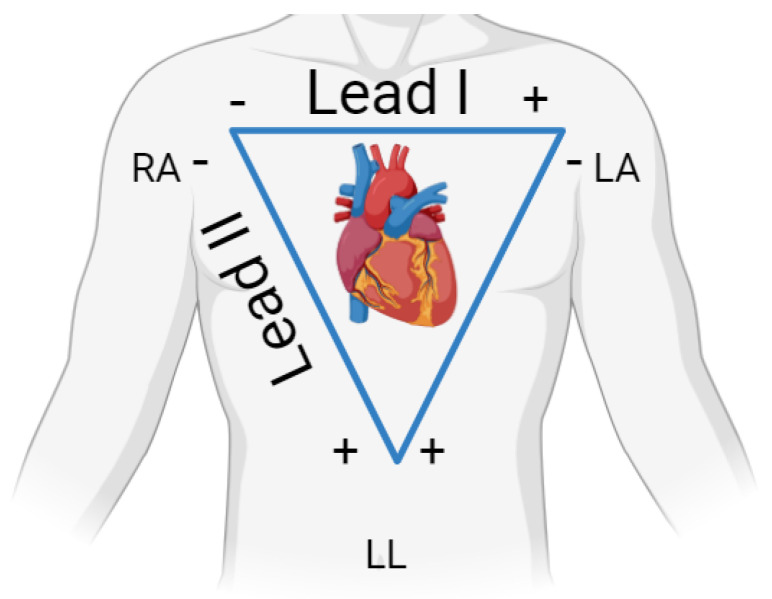
Electrode placement for ECG Lead I and Lead II, forming Einthoven’s triangle.

**Figure A2:**
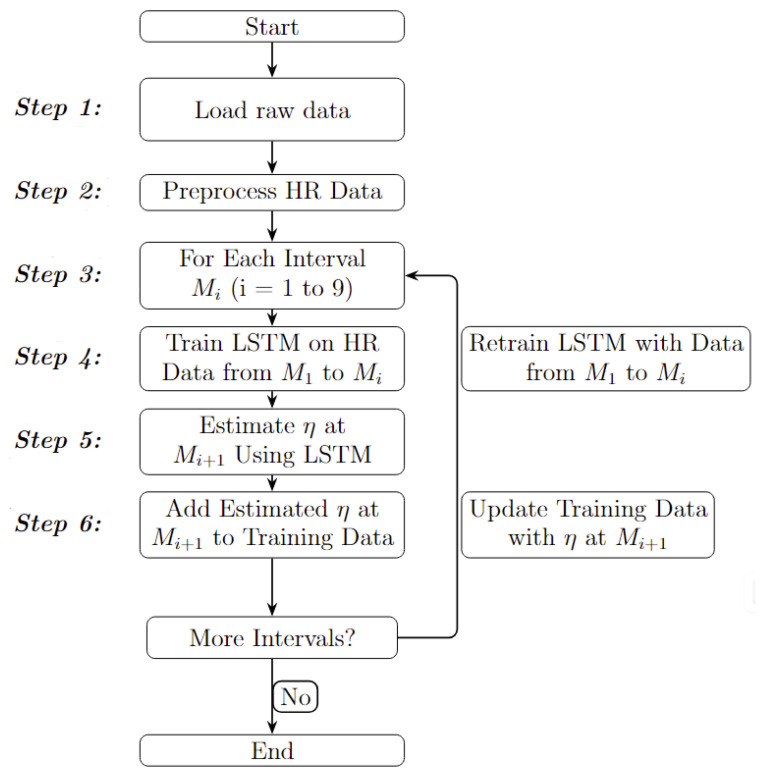
Flowchart illustrating the estimation mechanism used to predict the tissue hysteresivity coefficient η.

**Figure A3:**
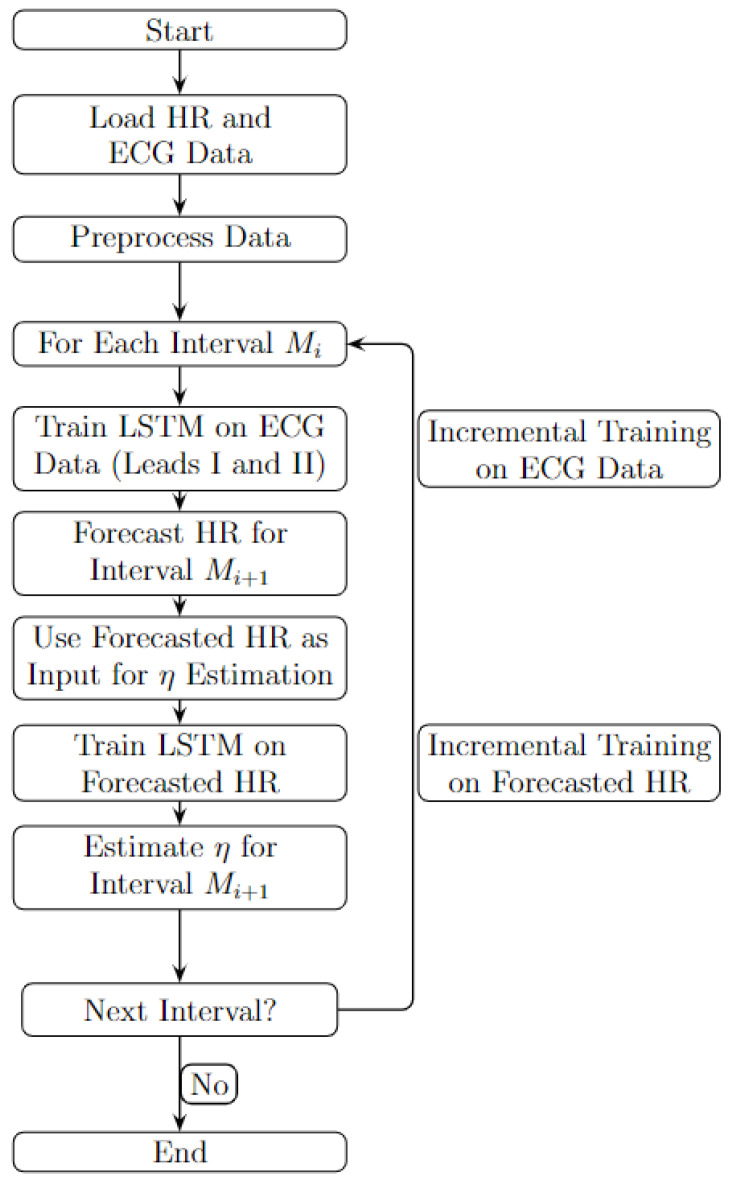
Flowchart illustrating the forecasting mechanism used to predict the tissue hysteresivity coefficient η.

## Data Availability

Data are contained within the article.

## Appendix A. Calculation of the *η* Parameter

The η parameter, also known as the hysteresivity coefficient η, is derived from the identified model parameters of the respiratory system using fractional-order models. This appendix provides a detailed description of the steps involved in calculating η [10].

The fractional-order model for the human respiratory system provides an expression for the input impedance ($Z_{r}\left(s\right)$), measured at the mouth of the subject. This model considers a series arrangement of resistance ($R_{r}$), inertance ($L_{r}$), and a parameter $D_{r}$, which is the reciprocal of the compliance ($C_{r}$):

$$Z_{r}\left(s\right)=R_{r}+L_{r}s^{\alpha_{r}}+\frac{D_{r}}{s^{\beta_{r}}}$$

where $R_{r}$ is the airway resistance in kPa/(l/s); $L_{r}$ is the inertance in kPa/(l/s^2^); $D_{r}=\frac{1}{C_{r}}$, where $C_{r}$ is the compliance in l/kPa; $\alpha_{r}$ and $\beta_{r}$ are fractional orders; and *s* is the Laplace operator.

The model parameters ($R_{r}$, $L_{r}$, $C_{r}$, $\alpha_{r}$, $\beta_{r}$) are identified using genetic algorithms. Genetic algorithms are heuristic optimization techniques inspired by the process of natural selection, which iteratively improve potential solutions to optimization problems [36,37,38].

From the identified model parameters, tissue damping ($G_{r}$) and tissue elastance ($H_{r}$) are calculated using the following equations:

$$G_{r}=\frac{1}{C_{r}\omega^{\beta_{r}}}\cos\left(\beta_{r}\frac{\pi }{2}\right)$$

$$H_{r}=\frac{1}{C_{r}\omega^{\beta_{r}}}\sin\left(\beta_{r}\frac{\pi }{2}\right)$$

where ω is the angular frequency.

The hysteresivity coefficient η is then defined as the ratio of tissue damping ($G_{r}$) to tissue elastance ($H_{r}$):

$$\eta_{r}=\frac{G_{r}}{H_{r}}$$

This parameter characterizes the heterogeneity of lung tissue and has been shown to vary significantly with pathology.

## Appendix B. LSTM Model Architecture

The LSTM model operates on a training dataset consisting of input sequences $\left\{x_{t}\right\}$ and corresponding output sequences $\left\{y_{t}\right\}$, with *t* denoting the time steps. The LSTM model is composed of the following layers:

1. Sequence Input Layer: This layer accepts the input data and initializes the sequence for LSTM processing. Each time step *t* passes the feature vector $x_{t}$ to the subsequent layers.

2. LSTM Layer: The core of the model, the LSTM layer, operates with mathematical equations and maintains internal variables, including the following:

- Input Gate ($i_{t}$): The input gate controls the flow of new information into the cell state. It is computed using the sigmoid activation function σ and is defined as
  (A1) $$i_{t}=\sigma \left(W_{xi}x_{t}+W_{hi}h_{t-1}+b_{i}\right).$$
- Forget Gate ($f_{t}$): The forget gate controls the retention of past information in the cell state and acts as a weighting factor for past-to-new data. It is computed similarly to the input gate and is defined as
  (A2) $$f_{t}=\sigma \left(W_{xf}x_{t}+W_{hf}h_{t-1}+b_{f}\right).$$
- Candidate Cell State (${\tilde{C}}_{t}$): The candidate cell state represents the new information that could be stored in the cell state. It is computed using the hyperbolic tangent activation function and is defined as
  (A3) $${\tilde{C}}_{t}=\tanh\left(W_{xc}x_{t}+W_{hc}h_{t-1}+b_{c}\right).$$
- Cell State Update ($C_{t}$): The cell state $C_{t}$ is updated by combining the previous cell state $C_{t-1}$ with the new information from the input gate and candidate cell state:
  (A4) $$C_{t}=f_{t}\cdot C_{t-1}+i_{t}\cdot {\tilde{C}}_{t}.$$
- Output Gate ($o_{t}$): The output gate controls what information from the cell state should be used to compute the output. It is defined as

(A5) $$o_{t}=\sigma \left(W_{xo}x_{t}+W_{ho}h_{t-1}+b_{o}\right).$$

Here, σ represents the sigmoid activation function, tanh is the hyperbolic tangent activation function, *W* represents weight matrices, *b* represents bias vectors, and $h_{t-1}$ is the previous hidden state.

3. Fully Connected Layer: Following the LSTM layer, the fully connected layer prepares the data for the final output by applying the learned weights and biases to the output of the LSTM layer.

4. Regression Layer: The final regression layer assigns a continuous value to each time step, ensuring accurate prediction of the output sequence.

## Appendix C. ECG Lead I and Lead II Placements

Electrocardiography (ECG) is a method used to measure the electrical activity of the heart. ECG Lead I and Lead II are two of the standard limb leads used in this measurement. The electrode placements and the relationship between these leads are essential for accurate cardiac assessment.

ECG Lead I measures the potential difference between the left arm (LA) and right arm (RA) electrodes. This lead provides valuable information about the heart’s electrical activity in the horizontal plane and is useful for detecting abnormalities in the lateral part of the heart [39]. ECG Lead II measures the potential difference between the right arm (RA) and left leg (LL) electrodes. This lead is crucial for identifying the rhythm of the heart and is commonly used in monitoring and diagnosing arrhythmias [40]. The electrodes are placed in the following positions:

- Right Arm (RA): Electrode placed on the right arm.
- Left Arm (LA): Electrode placed on the left arm.
- Left Leg (LL): Electrode placed above the left ankle.

The relationship between Lead I and Lead II is defined by Einthoven’s triangle, which is a geometrical representation of the limb leads’ configuration [41]. Einthoven’s triangle helps in understanding the heart’s electrical activity from different angles and is essential for interpreting the overall cardiac function.

Figure A1 illustrates the electrode placement for ECG Lead I and Lead II, forming Einthoven’s triangle. The relationship between Lead I and Lead II is defined by this triangle, which is a geometrical representation of the limb leads’ configuration [41]. Einthoven’s triangle helps in understanding the heart’s electrical activity from different angles and is essential for interpreting overall cardiac function.

## Appendix D. Estimation Mechanism Used to Predict *η*

The mechanism for estimating the tissue hysteresivity coefficient η is presented in the flowchart in Figure A2. It outlines the process used to estimate η based on HR data by integrating a Long Short-Term Memory (LSTM) model.

The process illustrated in the flowchart comprises several key steps to estimate the tissue hysteresivity η using LSTM. In Step 1, the raw data are loaded. Step 2 involves preprocessing the HR data, where any outliers are identified and smoothed. Additionally, the HR data are normalized by scaling them to have a mean of 0 and a standard deviation of 1, which ensures the data are standardized for better performance during model training.

Step 6 involves adding the newly estimated η value from interval $M_{i+1}$ to the training dataset, which ensures that the model is continuously updated with the most recent data. Finally, the process checks whether there are more intervals to process. If there are, the LSTM model is retrained with the updated data, and the cycle repeats. This iterative approach allows the model to improve with each new estimation, refining the accuracy of η predictions with each additional interval. In Step 3, the process proceeds by iterating through different intervals $M_{i}$ (where i=1 to 9). Here, the LSTM model is trained on the HR data from the first interval $M_{1}$ up to the current interval $M_{i}$. Steps 4 and 5 involve using the trained LSTM model to estimate the η value at the subsequent interval $M_{i+1}$.

The *Deep Learning* Toolbox in MATLAB^®^ R2022a from MathWorks^®^ was used for the implementation of the LSTM network, including the layers (sequenceInputLayer, lstmLayer, fullyConnectedLayer, regressionLayer) and training functions (trainNetwork, predict) [42]. The network architecture begins with a sequenceInputLayer that processes sequences of heart rate (HR) data, followed by an lstmLayer with 50 hidden units, using sigmoid and tanh activation functions for gate control and state scaling. A fullyConnectedLayer maps the LSTM outputs to the desired η values, and a regressionLayer produces the final forecast. The model is trained using the Adam optimizer with a learning rate of 0.005 over 80 epochs. This architecture was selected to balance model complexity and the ability to capture temporal dependencies in the HR data, informed by a combination of trial and error and established practices in time-series forecasting.

## Appendix E. Forecasting Mechanism Used to Predict *η*

The forecasting mechanism used in this study integrates the training and forecasting of HR and the subsequent estimation of the tissue hysteresivity coefficient η using LSTM networks.

As shown in Figure A3, the process illustrated in the flowchart comprises several key steps to forecast and estimate the tissue hysteresivity coefficient η using LSTM networks. In Step 1, the heart rate (HR) and electrocardiogram (ECG) data are loaded into MATLAB. Step 2 involves preprocessing these data, where any outliers are identified and smoothed. The data are also normalized by scaling them to have a mean of 0 and a standard deviation of 1, ensuring a standardized input for model training.

In Step 3, the process begins by iterating through different intervals $M_{i}$ (where i=1 to 9). In Step 4, an LSTM model is trained on the ECG data (specifically using Leads I and II) to forecast the HR for the next interval $M_{i+1}$. Steps 5 and 6 provide the forecasted HR, which is used as input for the next stage of η estimation. Steps 7 and 8 use the forecasted HR to train another LSTM model that estimates the η value for interval $M_{i+1}$. Finally, the process involves adding this newly estimated and forecasted data to the training sample, allowing for incremental updates to the model. The process checks whether there are more intervals to process. If there are, the models are retrained with the updated data, and the cycle repeats. This iterative approach ensures that the models continuously improve, enhancing the accuracy of the forecasts and estimates for η.

The *Deep Learning* Toolbox in MATLAB^®^ R2022a from MathWorks^®^ was used to implement the LSTM-based forecasting model. The network architecture consists of two lstmLayer layers, each with 150 hidden units, designed to capture the temporal dependencies in the ECG data derived from the Lead I and Lead II placements. The lstmLayer is bidirectional, processing data in both forward and backward directions, which enhances the model’s ability to understand complex patterns in the time series. The network also includes a fullyConnectedLayer, which maps the outputs of the LSTM layers to a single output neuron corresponding to the predicted HR for the next time step. A regressionLayer is used to compute the loss between the predicted and actual HR values during training. The model is trained using the Adam optimizer, and the training is performed over 150 epochs. This LSTM architecture was chosen based on its ability to effectively model the sequential nature of ECG data, enabling accurate HR forecasting, which is then used to estimate the hysteresivity coefficient η, as described in this study.
